# Signaling Crosstalk between PPAR**γ** and BMP2 in Mesenchymal Stem Cells

**DOI:** 10.1155/2012/607141

**Published:** 2012-12-20

**Authors:** Ichiro Takada, Yoshiko Yogiashi, Shigeaki Kato

**Affiliations:** ^1^Department of Cell and Tissue Biology, School of Medicine, Keio University, Tokyo 160-8582, Japan; ^2^Department of Microbiology and Immunology, School of Medicine, Keio University, Tokyo 160-8582, Japan; ^3^Institute of Molecular and Cellular Biosciences, University of Tokyo, Tokyo 113-0032, Japan

## Abstract

Recent studies have revealed that PPAR**γ**'s transactivation function is regulated by extracellular signals. In particular, cytokines and Wnt family proteins suppress the ligand-inducible transactivation function of PPAR**γ** and attenuate adipogenesis/osteoblastogenesis switching in mesenchymal stem cells (MSCs). For example, Wnt5a suppresses PPAR**γ** transcriptional activity through the NLK/SETDB1/CHD7 pathway. Among these factors, BMP2 strongly induces bone formation, but the effect of BMP2 on PPAR**γ** function remains unclear. We examined the effect of BMP2 and PPAR**γ** in ST2 cells and found that PPAR**γ** activation affected BMP2's signaling pathway through epigenetic regulation. Although BMP2 did not interfere with PPAR**γ**-mediated adipogenesis, BMP2 increased mRNA expression levels of PPAR**γ** target genes (such as *Fabp4* and *Nr1h3*) when cells were first treated with troglitazone (TRO). Moreover, PPAR**γ** activation affected BMP2 through enhancement of histone activation markers (acetylated histone H3 and trimethylated Lys4 of histone H3) on the *Runx2* promoter. After TRO treatment for three hours, BMP2 enhanced the levels of active histone marks on the promoter of a PPAR**γ** target gene. These results suggest that the order of treatment with BMP2 and a PPAR**γ** ligand is critical for adipogenesis and osteoblastogenesis switching in MSCs.

## 1. Introduction

Mesenchymal stem cells (MSCs) are useful tools for regeneration therapy because of the ease of their isolation from patients and straightforward handling in culture. MSCs are derived from various adult tissues (such as adipose tissue and bone marrow) and have the potential to differentiate into a variety of lineages, including osteoblasts, chondrocytes, adipocytes, and myocytes [1, 2]. Recent studies have identified differentiation regulators in MSCs. Among these factors, PPAR*γ* is commonly accepted as the master adipogenic factor since activation of PPAR*γ* in precursors of nonadipogenic lineage cells triggers their transdifferentiation into adipocytes [3, 4]. Endogenous and synthetic PPAR*γ* agonists (15-deoxy-Δ(12,14)-prostaglandin J2 and thiazolidinediones) promote adipogenesis and inhibit osteoblastogenesis in primary bone marrow MSC culture [5]. Moreover, treatment of mice with rosiglitazone (a thiazolidinedione) increases bone marrow adiposity and decreases bone mineral density (BMD, or bone mass) apparently through suppression of pro-osteoblastic transcription factors *Runx2*, *Osterix,* and *Dlx5 *[6, 7]. Haploinsufficiency of PPAR*γ* in mice results in enhanced osteoblastogenesis and decreased bone marrow adipogenesis with increased trabecular bone volume [8]. Similarly, PPAR*γ* ^*hyp*/*hyp*^ mice, which carry hypomorphic mutations in the PPAR*γ* loci and show reduced expression of both PPAR*γ* isoforms in white adipose, show abnormally increased bone formation leading to insufficient space in the marrow cavity to maintain normal hematopoiesis [9]. Collectively, these *in vivo *and *in vitro *studies suggest that in the bone marrow, PPAR*γ* functions as a differentiation switch between osteoblastogenesis and adipogenesis, acting as an inhibitor of osteoblastogenesis. However, the molecular basis of such inhibitory action of PPAR*γ* in osteoblastogenesis is poorly understood. Specifically, it is important to understand the transactivation function of PPAR*γ*, as it is critical for regulating the switching between adipogenesis and osteoblastogenesis.

In addition to ligand-dependent activation, various signaling pathways can modulate the transactivation function of PPAR*γ* in MSCs. For example, several cytokines (TGF*β*1 and BMP2) inhibit adipocyte differentiation from bone marrow MSCs through suppression of PPAR*γ* transactivation function [10, 11]. Furthermore, Wnt ligands regulate differentiation of MSCs into osteoblasts and adipocytes [12]. Canonical Wnt signals (Wnt1, Wnt3a, Wnt5b, Wnt7a, Wnt10b, etc.) promote bone formation, as initially illustrated in clinical studies of the Wnt receptor subunit gene, *LRP5*, and gain-of-function mutations [11, 13–17]. Another group of Wnt ligands (Wnt4, Wnt5a, and Wnt11, etc.) activates non-canonical Wnt signaling through cell membrane heterodimers of Fzd and Ror1 and Ror2 [18] and increases bone formation. We previously found that Wnt5a appeared to switch MSC differentiation fate from adipogenesis to osteoblastogenesis via suppression of PPAR*γ* function through the NLK/SETDB1/CHD7 complex [19]. Interestingly, *Setdb1* mRNA was suppressed by PPAR*γ* during adipogenesis [20] and Wnt5a regulated osteoclast differentiation via control of *Tnfsf11* mRNA expression levels in osteoblasts [21]. 

 Bone morphogenetic proteins (BMPs) are cytokines that induce the differentiation of osteoprogenitor cells to osteogenic cells and have the potential to act as autologous bone graft substitutes [22]. In particular, BMP2 is highly osteoinductive, inducing bone formation by stimulating the differentiation of mesenchymal cells into chondroblasts and osteoblasts. Recent clinical trials have shown that therapy using recombinant human BMP2 offers significant clinical potential [23]. On a molecular level, skeletal progenitor cells lacking BMP2 have reduced mRNA levels of *Sp7*, *Wnt1*, *Lrp5*, *Fzd1*, *Axin1,* and *Axin2* [24]. However, the effects of BMP2 on the transactivation function of PPAR*γ* and adipogenesis remain unclear. In this study, we analyzed signaling crosstalk between PPAR*γ* and BMP2 in MSCs. 

## 2. Methods

### 2.1. Cell Culture and Adipocyte and Osteoblast Differentiation

ST2 stromal cells derived from mouse bone marrow were cultured in *α*MEM medium containing 10% fetal bovine serum (FBS) and penicillin/streptomycin (Life Technologies) at 37°C and 5% CO_2_. For adipocyte differentiation, ST2 cells were treated with/without troglitazone (one *μ*M) and/or BMP2 (50 ng/mL). After seven days, lipid accumulation was assessed by staining with Oil-Red-O. In brief, cells were washed with phosphate buffered saline (PBS; 137 mM NaCl, 2.7 mM KCl, 10 mM Na_2_HPO_4_, 1.76 mM KH_2_PO_4_) and fixed with 4% formaldehyde/PBS for five min at room temperature. After a PBS wash, the cells were stained for 15 min with Oil-red O in buffer (70% isopropanol/PBS that had been filtered). After staining, cells were washed with PBS twice and visualized by microscopy.

For ALPL staining, cells were washed with PBS and fixed with 4% formaldehyde/PBS. They were stained with a mixture of 0.1 mg/mL naphthol AS-MX phosphate (Sigma), 0.6 mg/mL fast-blue BB salt (Sigma), two mM MgCl_2_, five *μ*L/mL N,N-dimethylformamide (Wako), and 100 mM Tris-HCl (pH 8.8) buffer at 37°C for five to ten min. When the cells turned blue, the cells were washed twice with PBS and visualized by microscopy.

### 2.2. RT-qPCR Analysis

For reverse transcription-quantitative PCR (RT-qPCR), one *μ*g of total RNA from each sample was reverse transcribed into first-strand cDNA with random hexamers using Superscript III reverse transcriptase (Invitrogen). Primer sets for all genes were purchased from Takara Bio Inc. (Tokyo, Japan), including *Nr1h3,* MA056056; *Fabp4*, MA034980; *Runx2*, MA056487; *Alpl*, MA024599; *Gpd1*, MA066553; *Gapdh*, MA050371. Real-time RT-PCR was performed using SYBR Green SuperMix with the thermal cycler CFX96 (Bio-Rad) according to the manufacturer's instructions. Experimental samples were matched to a standard curve generated by amplifying serially diluted products using the same PCR protocol. To correct for variability in RNA recovery and the efficiency of reverse transcription, glyceraldehyde-3-phosphate dehydrogenase cDNA was amplified and quantified in each cDNA preparation. Normalization and calculation steps were performed according to the manufacturer's protocol.

### 2.3. ChIP Analysis

Chromatin immunoprecipitation (ChIP) analysis was performed using a ChIP assay kit (Millipore) according to the manufacturer's instructions. Soluble chromatin prepared from 1 × 10^6^ cells was immunoprecipitated with antibodies against the indicated proteins. For amplification of the Fabp4 promoter by qPCR, we used the primer pairs, 5′-TGCCCTCTCAGGTTTCATTTCT-3′ and 5′-AGTTGTGGTGGGTGGTTATGG-3′, for the Fabp4 gene promoter region at the PPRE. In addition, 5′-GCTCAGAACGCCACACACTC-3′ and 5′-TCTACCCCTCCTCCCTTTCC-3′ were used for the *Runx2* gene promoter region. For PCR, we used the primer pairs, 5′-AGTTC ACTAGTGGAAGTGTCACAGC-3′ and 5′-CTAGAAACAGACACTGGAACCACTCT-3′, for the *Fabp4* gene promoter region at PPRE. PCR conditions for semi-quantitative measurement were 27 cycles of 30 sec at 96°C, 45 s at 56°C, and one min at 72°C. PCR products were visualized on 2% agarose-Tris-acetate-EDTA (TAE) gels. 

## 3. Results

### 3.1. BMP2 Did Not Interfere with PPAR*γ*-Dependent Adipogenesis

**Figure 1:**
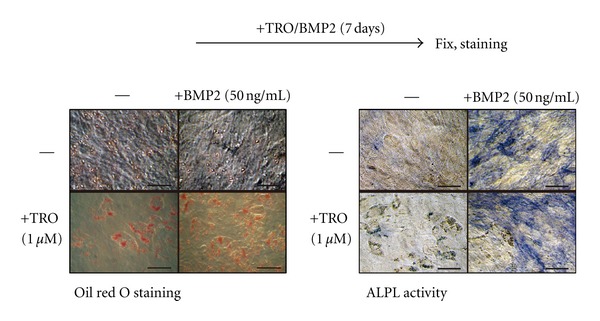
The effect of BMP2 and TRO on adipocyte and osteoblast differentiation of ST2 cells. The scheme of experiments are shown in upper panel. ST2 cells were cultured with one *μ*M troglitazone (TRO) or 50 ng/mL human recombinant BMP2 for seven days. The cells were fixed with 4% formaldehyde/PBS for five min at room temperature, and stained with Oil red O or for alkaline phosphatase (ALPL) activity. Scale bar means 50 *μ*m.

To elucidate the molecular link between BMP2 and PPAR*γ*, we first examined the effect of BMP2 on adipocyte/osteoblast differentiation by analyzing the response of ST2 cells. These cells are derived from mouse bone marrow stromal cells and can differentiate into adipocytes or osteoblasts [25]. We treated ST2 cells with one *μ*M troglitazone (TRO) as a PPAR*γ* ligand and/or 50 ng/mL BMP2 to induce adipocytes or osteoblasts. We confirmed adipocyte differentiation by Oil red O staining and osteoblasts by staining for alkaline phosphatase (ALPL) activity. As previously reported, TRO induced adipogenesis but not osteoblastogenesis. However, ST2 cells treated with both TRO and BMP2 for seven days underwent adipogenesis and osteoblastogenesis (Figure 1). These results show that PPAR*γ* and BMP2 do not interfere with one another.

**Figure 2:**
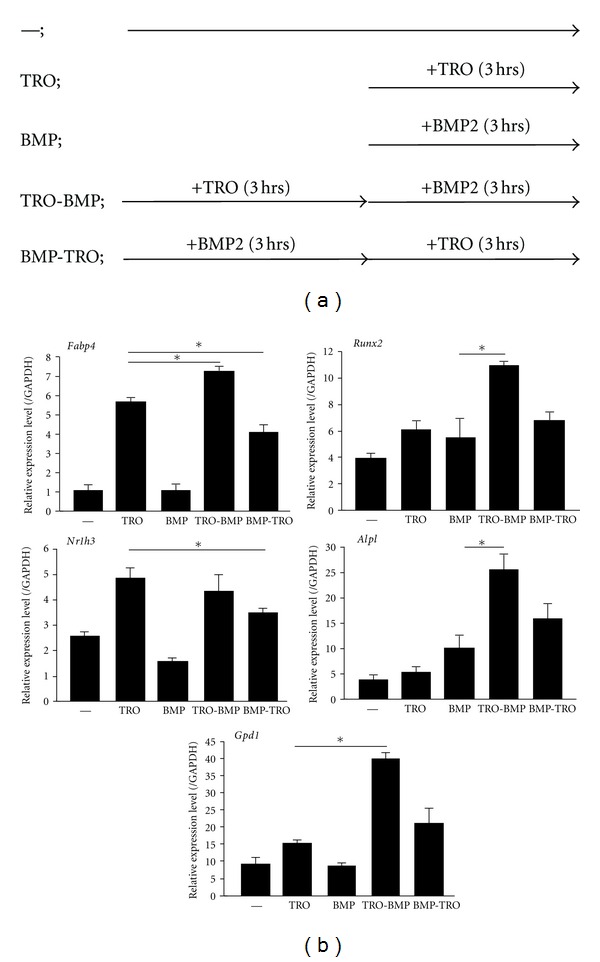
Adipocyte and osteoblast marker gene mRNA levels were altered by changing the order of BMP2 and TRO treatment. (a) Scheme of experiments used in Figures 2 and 3. We examined five conditions. (-) Untreated ST2 cells were cultured in *α*MEM supplemented with 10% FBS and penicillin/streptomycin. (TRO) ST2 cells were incubated with one *μ*M TRO for three hr. (BMP) ST2 cells were treated with 50 ng/mL BMP2 for three hr. (TRO-BMP) ST2 cells were treated with TRO for three hr, the medium was changed, and the cells were then incubated with BMP2 for three hr. (BMP-TRO) ST2 cells were treated with BMP2 for three hr and the medium was changed; the cells were then incubated with TRO for three hr. (b) RT-qPCR analyses of adipocyte or osteoblast differentiation marker genes. After ST2 cells were cultured under the conditions described above, cells were collected and RNAs were extracted. Then RT-qPCR experiments were performed and normalized to *Gapdh* mRNA. Each experiment was performed at least three times. Student's *t*-test was performed. *P < 0.05.

**Figure 3:**
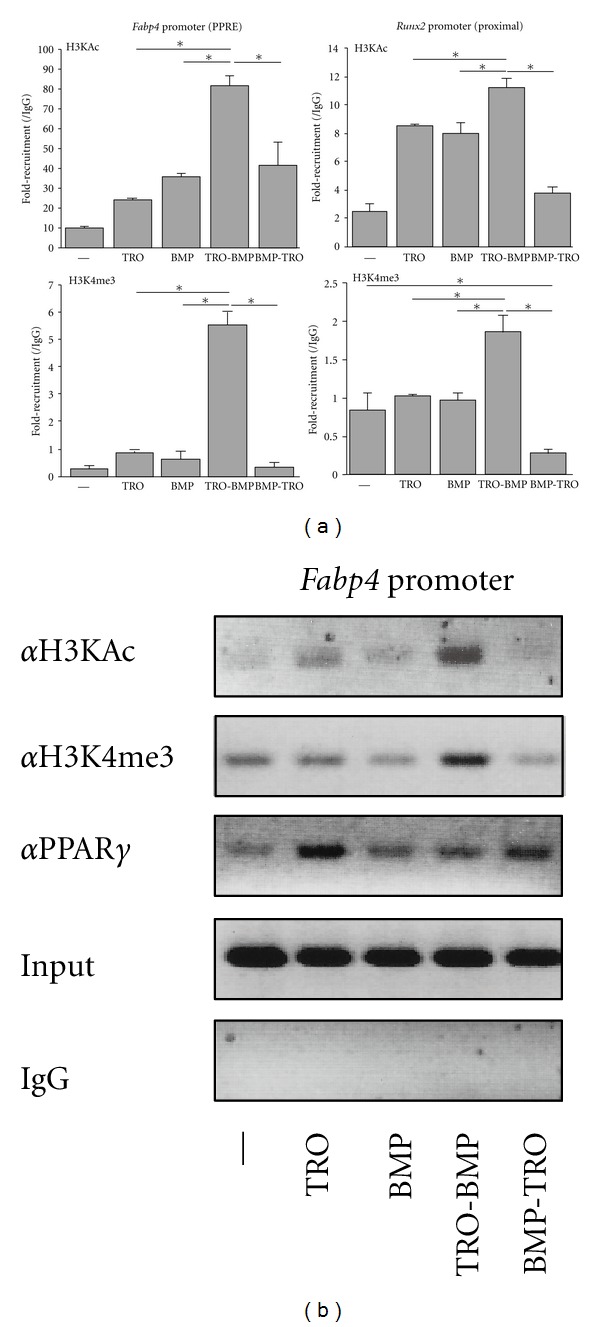
ChIP analysis of ST2 cells treated with/without BMP2 and/or TRO. (a) After ST2 cells were cultured under the conditions described in Figure 2, cells were lysed and ChIP analysis was performed according to the manufacturer's protocol (Millipore) using the indicated antibodies. H3KAc, anti-acetylated Lys histone H3 (Millipore); H3K4me3, anti-trimethylated Lys4 histone H3 (Millipore). Samples were examined by qPCR using CFX96 (Bio-Rad). Primers were used as previously reported [19]. Each experiment was performed at least three times. Student's *t*-tests were performed. *P < 0.05. (b) ChIP analysis of ST2 cells using anti-H3AcK, -H3K4me3, and -PPAR*γ*. ChIP assays were performed under the same conditions as (a). PCR was performed as described and visualized on 2% agarose TAE gels.

BMP2 reportedly inhibits PPAR*γ*-mediated adipogenesis [26]; furthermore, BMP2-dependent osteoblastogenesis is suppressed by PPAR*γ* activation [27]. These reports contradicted our findings. We hypothesized that the order of treatment with BMP2 and PPAR*γ* ligand may attenuate the activation of one or the other differentiation pathway. Thus, we treated ST2 cells with BMP2 and then TRO or with TRO and then BMP2 for three hr each (Figure 2(a)). We then performed RT-qPCR and ChIP analysis (Figure 2(b) and Figures 3(a) and 3(b)). Interestingly, we found that the expression levels of adipocyte or osteoblast marker genes varied according to the order in which TRO and BMP2 were added to the cells. For example, after treatment with BMP2 for three hr, TRO-dependent induction of *Fabp4* and *Nr1h3* mRNAs (PPAR*γ* target genes in adipocytes [4, 28]) was repressed in ST2 cells (Figure 2(b) left, compare TRO (three hr) with BMP2 (three hr)-TRO (three hr)). Interestingly, after TRO treatment for three hr, BMP2 induced mRNA levels of adipocyte marker genes (*Fabp4* and *Gpd1*). In addition, pretreatment with TRO for three hr enhanced BMP2-dependent *Runx2* and alkaline phosphatase (*Alpl*) mRNA expression (Figure 3(b) right: compare BMP2 (three hr) with TRO (three hr)-BMP2 (three hr)). However, pre-treatment with BMP2 did not enhance TRO-dependent induction of their mRNAs (Figure 2(b)). These results indicated that PPAR*γ* activation enhanced BMP2 activity and pretreatment with PPAR*γ* ligand attenuated osteoblast-related gene expression.

### 3.2. Epigenetic Crosstalk between BMP2 Signaling and PPAR*γ*

Many transcriptional coactivators and corepressors of PPAR*γ* have been identified [29], some of which have histone modifying activity [30]. Histone modifications play pivotal roles in transcription by tightening or enlarging nucleosomes. The nucleosome is the fundamental unit of chromatin structure, and it consists of two copies of each of the core histones, H2A, H2B, H3, and H4, with DNA wrapped around the octameric core. The H3 and H4 histones possess N-terminal tails and are particularly susceptible to posttranslational modifications by specific enzymes. Modifications of chromatin via histone acetylation, methylation, and phosphorylation constitute important mechanisms of epigenetic regulation. Combinations of these modifications alter local chromatin structure or physical properties of histones. Thus, these changes can facilitate or block the transcriptional machinery and its ability to bind to the promoter and modulate activity. In fact, a number of the enzymes involved in histone modification can also alter non-histone proteins to influence transcription. Epigenetic enzymes that play pivotal transcriptional roles include histone methyltransferases (HMTs), histone demethylases (HDMs), histone acetyltransferases (HATs), histone deacetylases (HDACs), DNA methyltransferases, and DNA demethylases.

In general, trimethylation of lysine 4 of H3 (H3K4me3) and acetylation of lysine H3 (H3KAc) are considered transcriptionally active chromatin marks, whereas di- and tri-methylation of lysine 9 or 27 of H3 (designated H3K9me2, H3K9me3, H3K27me2, or H3K27me3) are marks of transcriptionally repressive chromatin. Epigenetic regulators underlie many activities of the nuclear receptor superfamily including PPAR*γ*. Therefore, studying epigenetic control by PPAR*γ* is key to understanding ligand-mediated transcriptional regulation of metabolic pathways.

To investigate whether the treatment order of TRO and BMP2 caused epigenetic changes, we performed qPCR ChIP analysis of the promoters of *Fabp4* or *Runx2*. As shown in (Figure 3(a)), in the Runx2 promoter region, higher levels of the transcriptionally active marks (histones H3KAc and H3K4me3) were induced when first treated with TRO and then with BMP2 than with BMP2 alone or TRO alone. Moreover, BMP2 pretreatment suppressed TRO-dependent induction of H3K4me3 on the *Fabp4* promoter. Interestingly, three hours of BMP2 treatment or three hr of TRO treatment highly suppressed H3K4me3 levels on the *Runx2* promoter compared to untreated cells. However, the recruitment of PPAR*γ* was not enhanced on the *Fabp4* promoter when treated with TRO (three hr)-BMP2 (three hr) (Figure 3(b)). These results showed that the order of BMP2 or TRO treatment attenuated transcription-related histone marks on adipocyte- or osteoblast-related gene promoters in MSCs.

## 4. Discussion

In this study, we found that the order of TRO or BMP2 treatment attenuated mRNA expression of adipogenesis/osteoblastogenesis-related genes in MSCs. In particular, treatment with TRO (three hr)-BMP2 (three hr) more induced mRNA levels of both adipogenesis (*Fabp4* and *Gpd1*) and osteoblastogenesis (*Runx2* and *Alpl*) related genes than TRO, BMP, or BMP (three hr)-TRO (three hr). These results may show that the activation of PPAR*γ* affects on BMP2-induced osteoblastogenesis. Moreover, on the *Fabp4* and *Runx2* promoters, transcriptionally active histone marks are more induced by TRO (three hr)-BMP2 (three hr) than TRO, BMP, or BMP (three hr)-TRO (three hr). These results coincide with each mRNA level (Figure 2(b)), and show that signaling crosstalk between PPAR*γ* and BMP2 are mediated by epigenetic regulation.

The underlying mechanisms guiding the lineage-specific differentiation of MSC are only partially understood. Improved understanding of these mechanisms could significantly enhance regeneration and transplantation therapy. In this manuscript, we showed that PPAR*γ* functions as a regulatory factor for BMP2 in MSCs. Moreover, it was recently shown that human MSC treated with PPAR*γ* inhibitor GW9662 and human MSC-derived extracellular matrix can repair bone with high efficiency [31]. Taken together, these studies show that, besides controlling the cells' environment, regulation of transcription and epigenetic modulation is required for appropriate bone regeneration. Epigenetic histone modification is particularly essential for suppression or activation of transcription. Although we found the epigenetic regulation-mediated signaling crosstalk between PPAR*γ* and BMP2, mechanism of PPAR*γ*-dependent enhancement of BMP2 activation remains unclear. It is possible that ligand-bound PPAR*γ* defines genome-wide positions of active transcriptional machinery during differentiation. Alternatively, direct interaction of ligand-bound PPAR*γ* and Smads could contribute to regulation of gene expression. Recent studies have shown that Smad2/3 determines cell type-specific responses by altering interacting transcriptional factors [32]. PPAR*γ* also may interact with Smads and attenuate mRNA expression levels of osteoblast marker genes. Further epigenetic studies and genome-wide analyses will be required to fully understand signaling crosstalk in MSCs.
